# Comprehensive Bioinformatics Analysis and Experimental Verification RNF186 Is a Recurrence Signature Gene of Hepatocellular Carcinoma that Promotes Cell Proliferation

**DOI:** 10.32604/or.2026.071617

**Published:** 2026-03-23

**Authors:** Shanbao Ke, Junya Yan, Xiao Feng, Baiyu Li

**Affiliations:** Department of Oncology, Henan Provincial People’s Hospital, Zhengzhou University People’s Hospital, Henan University People’s Hospital, Zhengzhou, China

**Keywords:** Hepatocellular carcinoma, single-cell RNA sequencing, recurrence, RNF186, cell proliferation

## Abstract

**Objectives:**

Tumor recurrence is a major determinant of poor prognosis in hepatocellular carcinoma (HCC), yet its cellular and molecular basis remains incompletely understood. This study aimed to identify recurrence-associated genes at single-cell resolution and to develop a prognostic model for predicting survival outcomes and immunotherapy responsiveness in HCC.

**Methods:**

Single-cell RNA sequencing data from 12 primary and 6 recurrent HCC samples were integrated and analyzed to identify genes characteristic of recurrence. After quality control, principal component analysis, and t-SNE-based clustering were used to identify highly variable genes for cell clustering and annotation. Based on macrophage characteristic genes, a recurrence-related risk score was constructed using a LASSO-Cox regression model, and a nomogram integrating clinical variables was developed. Prognostic performance was assessed using Kaplan–Meier analysis and time-dependent ROC curves. Immune infiltration profiling was performed to compare immune characteristics between risk groups defined by the prognostic model. Multivariate Cox regression was applied to identify independent prognostic biomarkers, which were subsequently validated by cell function experiments.

**Results:**

The risk model effectively stratified patients into high- and low-risk groups with distinct survival outcomes, demonstrating high predictive accuracy for 1-, 3-, and 5-year survival. High-risk patients showed altered immune profiles and a reduced predicted response to immunotherapy. GRID2, RNF186, and SLC4A10 were identified as independent prognostic genes, with RNF186 promoting HCC cell proliferation in a SESN2-dependent manner.

**Conclusion:**

This prognostic model provides new insights into precision medicine and immunotherapy for HCC, highlighting the potential clinical significance of RNF186 as a therapeutic target.

## 1 Introduction

Hepatocellular carcinoma (HCC) is the third leading cause of cancer-related deaths worldwide, accounting for 90% of primary liver cancer cases [1–3]. Despite advancements in treatment, including surgical resection and neoadjuvant therapy, approximately 70% of patients with resectable HCC experience relapse and metastasis, significantly limiting long-term survival [4–6]. Therefore, a deeper understanding of the molecular mechanisms underlying HCC progression and recurrence is essential for improving treatment strategies and developing personalized medicine.

Recurrence of HCC post-resection can be classified as early (within weeks or months) or late (more than two years post-resection) [7]. Early recurrence, which accounts for nearly 70% of all cases, is often attributed to micrometastases or incomplete tumor removal [8], whereas late recurrence is typically associated with newly formed primary tumors [4]. Ongoing clinical trials are exploring the efficacy of adjuvant immunotherapy to reduce recurrence risk after surgery [9]. However, molecular studies of HCC have largely focused on primary tumors, leaving the evolutionary relationship between primary and recurrent tumors poorly understood, with several biological and clinical questions remaining unanswered.

The emergence of next-generation sequencing (NGS) and single-cell sequencing technologies has led to the creation of extensive public tumor databases, enabling the integration and reanalysis of large datasets to uncover crucial insights into tumor pathogenesis [10,11]. Recently, prognostic models based on gene signatures have been increasingly adopted [12,13].

This study aimed to screen for prognostic features of recurrent hepatocellular carcinoma (HCC) by integrating multi-omics data and further strengthening the level of evidence through cellular function experiments.

## 2 Materials and Methods

### 2.1 Data Collection

**Figure 1:**
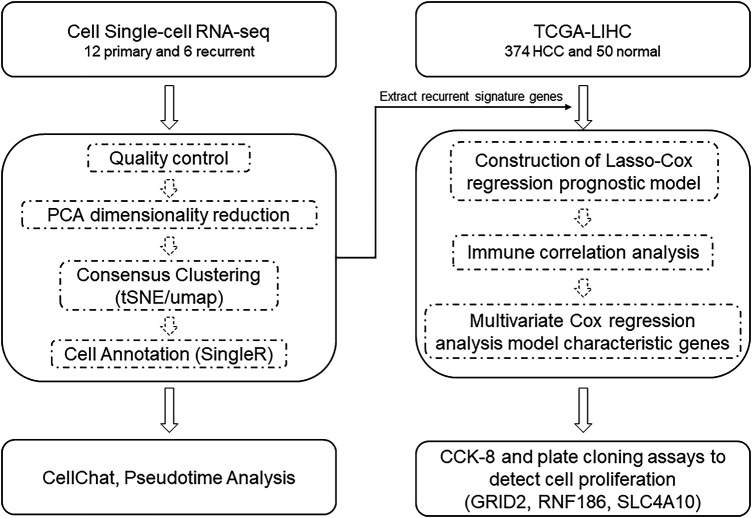
Work model. Abb: PCA: principal components analysis; tSNE: t-distributed stochastic neighbor embedding; UMAP: Uniform Manifold Approximation and Projection; TCGA: The Cancer Genome Atlas; HCC: Hepatocellular carcinoma; Cox: Cox regression model; CCK-8: Cell Counting Kit-8; GRID2: Glutamate Ionotropic Receptor Delta Type Subunit 2; RNF186: Ring Finger Protein 186; SLC4A10: Solute Carrier Family 4 Member 10.

The workflow is shown in Fig. 1. Single-cell transcriptome data from 12 primary hepatocellular carcinoma (HCC) tumors and 6 recurrent tumors were obtained from the study of Sun et al. [10]. 374 tumor samples and 50 adjacent normal samples were obtained from The Cancer Genome Atlas (TCGA) (https://portal.gdc.cancer.gov/, accessed on 02 September 2024), supplemented with gene expression information and clinical information, including overall survival, survival status, patient age, sex, and TNM stage, from the GTEx database (https://gtexportal.org/home/aboutAdultGtex, accessed on 23 October 2025). Additionally, ICGC-LIRI-JP (https://dcc.icgc.org/, accessed on 23 October 2025) and GSE14520 (https://www.ncbi.nlm.nih.gov/geo/query/acc.cgi?acc=GSE14520, accessed on 23 October 2025) were collected as independent validation cohorts. Batch effects were corrected using ComBat (from the sva package) during data merging. Clinical annotations included overall survival, survival status, patient age, sex, and TNM stage.

### 2.2 Single-Cell Sequencing Analysis

As the dataset had undergone prior preprocessing and quality control in the original publication, downstream analyses were performed directly using the Seurat (v5.0.2) R package [14]. Principal component analysis (PCA) followed by t-distributed stochastic neighbor embedding (t-SNE) was utilized for dimensionality reduction, and the Louvain algorithm was used for unsupervised clustering. Cellular identities were assigned with reference to the Human primary cell atlas (HPCA) in the SingleR (4.3.1) package [15].

### 2.3 Cell-Cell Interaction Network Analysis

After normalization and batch effect correction were performed using the NormalizeData function in the Seurat package, the CellChat v.1.6.1 R package was used to quantitatively infer and analyze intercellular communication networks based on scRNA-seq data, aiming to predict major cellular signal inputs and outputs and elucidate how cells and signaling coordinate various functions [16]. Signaling pathways were identified based on known ligand-receptor pairs (CellChatDB.human, https://github.com/sqjin/CellChat, accessed on 02 September 2024) and their expression in specific cell types. Adjust the key parameters of CellChat (such as the number of signal path groupings k value) to observe the stability of the results.

### 2.4 Risk Score Prognostic Model Construction

After standardizing and normalizing the gene expression data, the extracted differentially expressed genes associated with HCC recurrence were further screened using univariate Cox regression to enhance their prognostic relevance (*p* < 0.05). Subsequently, a multigene prognostic risk score was constructed using a minimum absolute contraction and selection operator (Lasso) regression model, and a nomogram was plotted by combining it with clinical parameters [17]. The reliability of the scores was assessed using calibration curves, Kaplan-Meier analysis, and the area under the time-dependent receiver operating characteristic (ROC) curve (AUC, 95% CI).

### 2.5 Functional Enrichment Analysis and Protein-Protein Interaction Network Construction

The functional enrichment analysis of genes was performed using the DAVID tool (version 6.8, https://davidbioinformatics.nih.gov/tools.jsp, accessed on 02 September 2024), with annotations for its terminology including Gene Ontology (GO) terminology and the Kyoto Encyclopedia of Genes and Genomes (KEGG) (https://www.kegg.jp/kegg/, accessed on 02 September 2024) (FDR < 0.05, log_2_ |Fold Change (FC)| > 2) [18,19]. The protein-protein interaction (PPI) network was constructed by uploading the gene list to the STRING database (version 11.0, https://string-db.org/, accessed on 02 September 2024) [20].

### 2.6 Evaluation of Immune Status, Tumor Stemness, and Genomic Heterogeneity

Three deconvolution algorithms—CIBERSORT, EPIC, and ESTIMATE—were used to comprehensively characterize immune cell infiltration, with expression matrices normalized and batch-effect corrected prior to each. The results from all three methods were analyzed to assess the overall immune status of the sample, rather than being limited to any single method [21,22]. Data quality control in calculating microsatellite instability (MSI) and tumor mutational burden (TMB) scores included excluding samples with incomplete mutation annotation, sequencing artifacts, or mutation counts exceeding three standard deviations of the cohort mean, while missing values were directly removed [23]. The Tumor Immune Dysfunction and Exclusion (TIDE) algorithm (http://tide.dfci.harvard.edu/, accessed on 02 September 2024) was used to predict the immune escape potential and response to immune checkpoint inhibitors in each subgroup [24].

### 2.7 Protein Structure Prediction and Mutation Analysis

Protein structures of the genes were accessed from the GeneCards database (https://www.genecards.org/, accessed on 02 September 2024). 296 samples with detected mutations were obtained from cBioPortal (https://www.cbioportal.org/, accessed on 02 September 2024), of which 153 (51.7%) were used for localization. The chi-square test was used to assess mutation frequency and variant types, including single nucleotide polymorphisms (SNPs), oligonucleotide polymorphisms (ONPs), insertions, and deletions, between the high-expression and low-expression groups.

### 2.8 Cell Culture and Transfection

Huh7 (CC0102) and PLC/PRF/5 (CC0111) cell lines were purchased from Guangzhou Saiku Biotechnology Co., Ltd. (Guangzhou, China). Both cell lines were authenticated by short tandem repeat (STR) profiling and confirmed to be free of mycoplasma contamination prior to use. Cells were cultured in a humidified incubator at 37°C in a supplemental medium (DMEM, Cat. No. 12491015, Thermo Fisher Scientific, Shanghai, China) containing 10% fetal bovine serum (FBS, Cat. No. C2739, Beyotime, Shanghai, China) and two antibiotics (penicillin 100 U/mL, Cat. No. ST486-10g, Beyotime and streptomycin 100 U/mL, Cat. No. C0222, Beyotime). For gene overexpression, SESN2 (NM_031459.5) and RNF186 (NM_019062.2) were generated by cloning human cDNAs into the pcDNA3-Flag vector. For gene silencing experiments, targeted small interfering RNAs (siRNAs) (BGI, Shenzhen, China) were transfected into cells using Lipofectamine 2000 reagent (Cat. No. 11668500, Thermo Fisher Scientific), following the manufacturer’s instructions. The final concentration of siRNA in the medium was adjusted to 20 nm. The specific siRNA sequences utilized are detailed in Table 1.

**Table 1:** siRNA sequence for gene.

Gene	Forward (5^′^-3^′^)	Reverse (5^′^-3^′^)
**GRID2#1**	UAAGAUCUCCUCAUUCUGGUU	CCAGAAUGAGGAGAUCUUACA
**GRID2#2**	ACAAACGUCACUGAAAAUGUG	CAUUUUCAGUGACGUUUGUUG
**GRID2#3**	UCUUGAACUGCUUGGAAAGGG	CUUUCCAAGCAGUUCAAGAAG
**RNF186#1**	UGUUGCAGGGUCUUGGUGCAG	GCACCAAGACCCUGCAACAGU
**RNF186#2**	UCACAUUCUGUGGAGCCAGAA	CUGGCUCCACAGAAUGUGACC
**RNF186#3**	AUUCUGUGGAGCCAGAAUGAC	CAUUCUGGCUCCACAGAAUGU
**SLC4A10#1**	UGUGUUUUGAGAAUAGAACGA	GUUCUAUUCUCAAAACACACU
**SLC4A10#2**	UCUCAAAGUGUGUUUUGAGAA	CUCAAAACACACUUUGAGAAA
**SLC4A10#3**	UUUCUCAAAGUGUGUUUUGAG	CAAAACACACUUUGAGAAAGA
**SESN2#1**	UUUCACGGCCUCGGAAGUCCG	GACUUCCGAGGCCGUGAAAAC
**SESN2#2**	AGCUUCUAGAACCCCGGGGCG	CCCCGGGGUUCUAGAAGCUCC
**SESN2#3**	UCCAGAACGCUCCGGAAUCCG	GAUUCCGGAGCGUUCUGGAGC

### 2.9 Western Blot Analysis

Forty-eight hours after siRNA transfection, total protein was extracted for immunoblot analysis. Cells were lysed in buffer containing 1% sodium dodecyl sulfate (SDS, Cat. No. ST626, Beyotime), and the total protein concentration was quantified using the bicinchoninic acid (BCA) assay kit (Cat. No. A55860, Thermo Fisher Scientific) according to the manufacturer’s protocol.

Equal amounts of protein (18 μg) lysate were separated by SDS-PAGE (10%) and transferred onto PVDF membranes (Cat. No. FFP19, Beyotime), followed by blocking with a 5% solution of skim milk (Cat. No. P0216-300g, Beyotime) powder dissolved in TBST at room temperature for 2 h. The membranes were incubated with primary antibodies in 4°C overnight targeting GRID2 (Dilution ratio, 1:1000, Cat. No. ab198499, Abcam, Shanghai, China), RNF186 (Dilution ratio, 1:1000, Cat. No. H00054546-M01, Biotechne, Wuhan, China), SLC4A10 (Dilution ratio, 1:1000, Cat. No. 27197-1-AP, Proteintech, Wuhan, China), HA (Dilution ratio, 1:5000, Cat. No. ab236632, Abcam), Flag (Dilution ratio, 1:2000, Cat. No. 20543-1-AP, Proteintech), SESN2 (Dilution ratio, 1:1000, Cat. No. ab178518, Abcam), or β-Actin (Dilution ratio, 1:3000, Cat. No. 66009-1-Ig, Proteintech) as a loading control. Secondary antibody incubation was performed using goat anti-rabbit (Dilution ratio, 1:2000, Cat. No. 27197-1-AP, Beyotime) or goat anti-mouse (Dilution ratio, 1:3000, Cat. No. 27197-1-AP, Beyotime) and incubated for 2 h at room temperature. Detection was performed using enhanced chemiluminescence (ECL, Cat. No. P0018S, Beyotime) reagents (Smart-Lifesciences) to visualize protein bands. All experiments were repeated three times.

### 2.10 Ubiquitination Assays and Co-Immunoprecipitation

Cells were cultured in 6 cm culture dishes, and during cell passage, the seeding density was maintained at at least 30% (usually one culture dish was passaged to three culture dishes) for ubiquitination analysis and immunoprecipitation experiments. When cell growth reaches a density of 60%–80%, cells were transfected according to the indicated conditions for 48 h and lysed using guanidine denaturing buffer (6 M guanidine hydrochloride, 0.1 M Na_2_HPO_4_/NaH_2_PO_4_, 10 mm imidazole, pH 8.0). After sonication, whole cell extracts were incubated with 30 μl anti-Flag magnetic beads (Cat. No. P2115, Beyotime) and washed, and the supernatant was immunoprecipitated at 4°C for 4 h or overnight (ensuring total protein content is above 1 mg). The immune complexes were washed three times with native lysis buffer, and the pulled-down proteins were analyzed by immunoblotting. For immunoprecipitation, cells were lysed using RIPA buffer (Cat. No. P0013, Beyotime) (non-denaturing lysis buffer). All other steps were the same as for the ubiquitination assay. All experiments were repeated three times. All immunoblot images were subjected to exposure analysis using Image Lab (v.3.0, Bio-Rad, Shanghai, China).

### 2.11 Real-Time Quantitative Polymerase Chain Reaction (RT-qPCR)

Total RNA was isolated from Huh7 cells using TRIzol (Cat. No. R0016, Beyotime, Shanghai, China) reagent following the standard protocol and using Exonuclease I (1 μl, Cat. No. 70073Z2500UN, Thermo Fisher Scientific) to avoid genomic contamination. First-strand complementary DNA (cDNA) synthesis was carried out using the iScript cDNA Synthesis Kit (Cat. No. 1708890, Bio-Rad, Hercules, CA, USA) using 20 ng of total RNA per replicate well. Quantitative PCR amplification was performed with Fast SYBR Green Master Mix (Bio-Rad, Hercules, CA, USA) on a real-time PCR system, using a three-step cycle of 35–40 times (95°C (15 s) + 60°C (15 s) + 72°C (45 s)). All experiments were biologically replicated three times.

Actin served as the internal control gene for normalization. Relative gene expression levels were calculated using the comparative 2^−ΔΔCt^ method. The primer sequences used in the analysis were as follows:

**GRID2-F:** 5^′^-TTTGTCCGTCTGGTGGTCTC-3^′^

**GRID2-R:** 5^′^-CAGTGCGAAATACCTCATCATCC-3^′^

**RNF186-F:** 5^′^-GGCTGGTGGATCCTGCTGAC-3^′^

**RNF186-R:** 5^′^-CTGCTCTCTGCAGAAGAGAG-3^′^

**SLC4A10-F:** 5^′^-GTCACAGGCATCGTGGTCATA-3^′^

**SLC4A10-R:** 5^′^-CCTTCACGCCAACAAATCTCAT-3^′^

**SESN2-F:** 5^′^-AGGACTACCTGCGGTTCGCC-3^′^

**SESN2-R:** 5^′^-GTAGTGCAGGCGCCAGAAGCTGG-3^′^

**Actin-F:** 5^′^-GACGGCCAGGTCATCACTATTG-3^′^

**Actin-R:** 5^′^-CCACAGGATTCCATACCCAAGA-3^′^

### 2.12 Colony Formation Assay

Approximately 1000 cells per well were plated into 6-well plates and cultured under standard conditions for a period of two weeks to allow colony development. Cells were fixed in 4% paraformaldehyde for 15 min at room temperature. Fixed cells were stained with 0.5% (w/v) crystal violet solution (Cat. No. C0775, Sigma-Aldrich, Shanghai, China) to visualize colonies. Images were acquired using the native camera of an iPhone 14 Pro. Clone counting was performed using ImageJ (v.1.53e, National Institutes of Health, Bethesda, MD, USA) software, following the official standard procedure.

### 2.13 Cell Viability Assay

Cell viability was assessed 48 h following siRNA transfection utilizing the Cell Counting Kit-8 (CCK-8, (Cat. No. C0037, Beyotime)). Briefly, cells were seeded in 96-well plates at a density of 2 × 10^4^ cells per well and allowed to adhere for 12 h. Each well then received 100 μl of complete DMEM medium along with 10 μl of the CCK-8 reagent, followed by incubation for 1 h at 37°C. Absorbance was measured at 450 nm using a Multiskan™ FC microplate reader (Cat. No. 1410101, Thermo Scientific, Waltham, MA, USA). All viability measurements were performed in triplicate to ensure reproducibility. Absorbance standardization: Five technical replicates were performed for each sample well, and the average value was taken as the final result. Standardization calculation: Sample value/Maximum sample value in this group.

### 2.14 Statistical Analysis

Comparative analyses between groups were conducted using the Wilcoxon rank-sum test, while Spearman’s rank correlation was applied to examine associations between variables. All statistical tests were two-tailed, with significance defined as *p* < 0.05. Analyses were conducted using R software (version 4.2.2).

## 3 Results

### 3.1 Single-Cell Analysis of Hepatocellular Carcinoma

**Figure 2:**
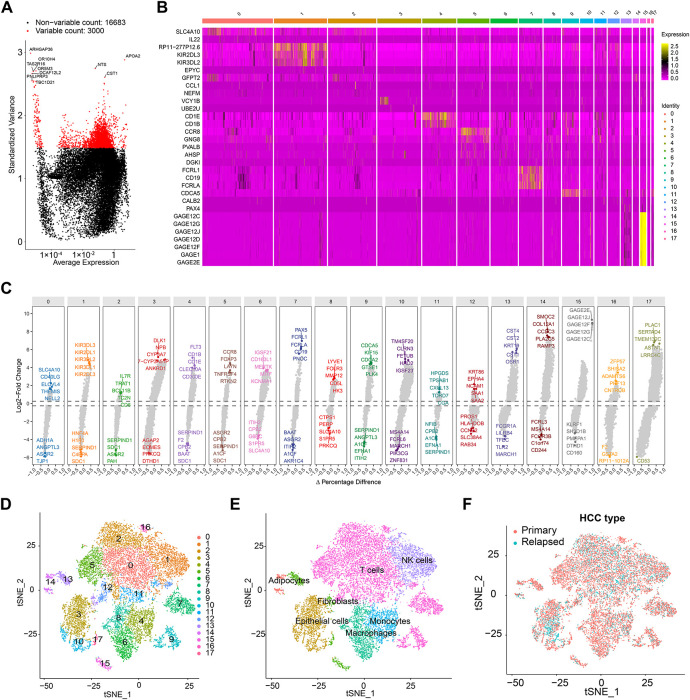

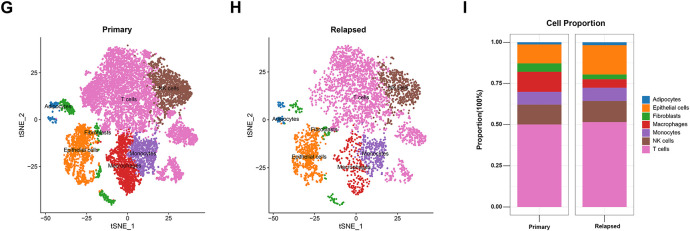
Single-cell sequencing analysis identifies relapse-related genes. (**A**) Volcano plot illustrating the top 3000 highly variable genes. (**B**) Heatmap showing the expression of key differential genes across different clusters. (**C**) Volcano plot displaying the top 10 key genes with the greatest differences across different cell types. (**D**) Principal component analysis (PCA) and t-SNE dimensionality reduction separating cells into 18 distinct clusters. (**E**) Cell annotation using the SingleR package, categorizing cells into 7 major groups. (**F**) t-SNE 2D plots colored by primary or recurrent samples. (**G**,**H**) t-SNE 2D plots of 12 primary samples (**G**) or 6 recurrent samples (**I**). (**I**) Proportion of cell content in 12 primary samples or 6 recurrent samples.

**Figure A1:**
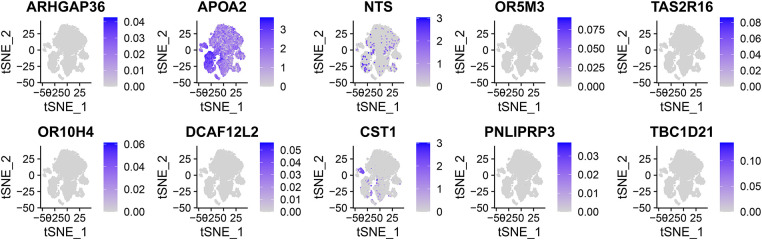
Expression distribution of the top 10 heterogeneous genes in single-cell t-SNE plot.

We obtained single-cell sequencing data from 18 hepatocellular carcinoma (HCC) samples, comprising 12 primary and 6 early-recurrent cases, as described by Sun et al. [10]. Using the “Seurat” package, we identified the top 3000 highly variable genes, which exhibited significant intercellular expression differences essential for cell-type identification (Fig. 2A). Principal component analysis (PCA) and t-SNE were employed for dimensionality reduction, clustering the samples into 18 distinct cell groups (Fig. 2B). Key differentially expressed genes were visualized via volcano plots (Fig. 2C). The top 10 heterogeneous genes were mapped in a t-SNE plot, showing cluster-specific expression, with APOA2 notably enriched (Fig. A1). Further annotation revealed seven major cell types: macrophages, T cells, NK cells, monocytes, endothelial cells, fibroblasts, and adipocytes (Fig. 2D,E). Comparison of t-SNE plots between primary and recurrent samples revealed minimal differences in overall cell distribution (Fig. 2F–H). However, recurrent samples displayed a notable reduction in macrophages and a significant increase in endothelial cells compared to primary samples (Fig. 2I).

### 3.2 Cell Communication Analysis and Identification of Relapse-Specific Genes

**Figure 3:**
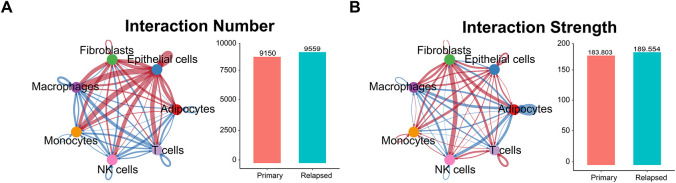

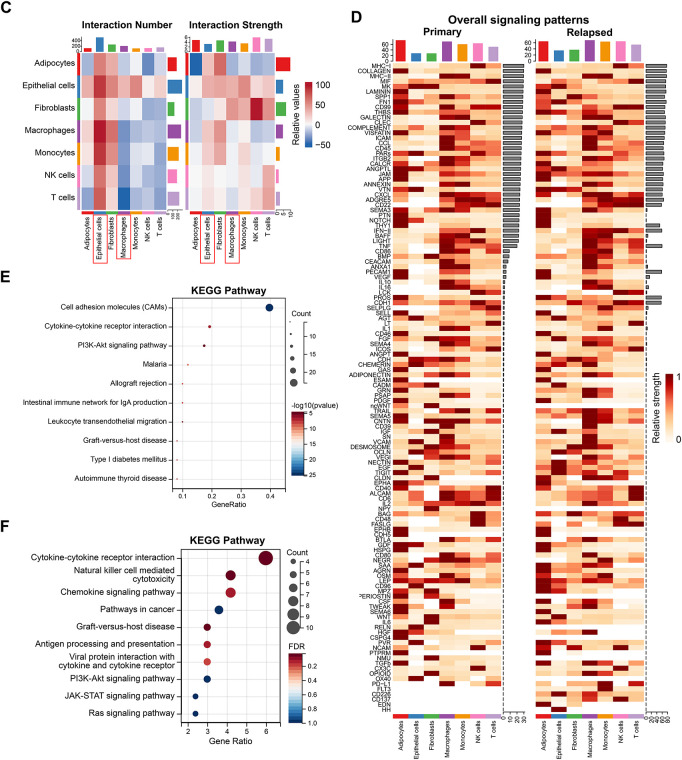
Extraction of relapse-specific genes and cell-cell communication. (**A**,**B**) Intercellular communication between 7 cell types. The left side is the communication network, the thickness represents the number of communications (**A**) or strength (**B**), and the color represents the direction of communication. The right side is the quantitative graph. (**C**) The number and strength of intercellular communication are represented by a heat map. The color depth represents the relative value. The left side is the number of communications, and the right side is the communication strength. The observed cell types are highlighted in the red box. (**D**) Gene expression heat map showing 126 differentially expressed genes between primary and relapse samples. (**E**) Kyoto Encyclopedia of Genes and Genomes (KEGG) analysis of 126 differentially expressed genes. (**F**) KEGG analysis of 180 recurrent signature genes.

**Figure A2:**
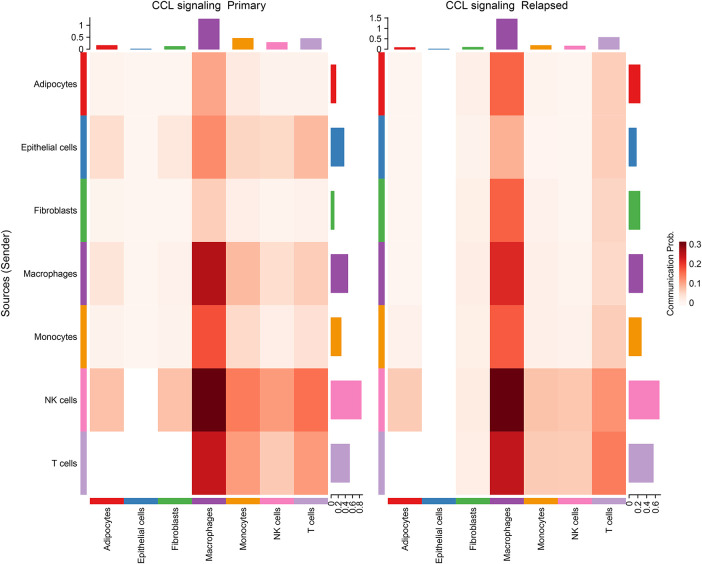
Heat map showing the differences in cell communication between chemoattractant cytokine ligand (CCL) signaling in primary and relapse samples.

Cell Chat analysis revealed an increase in both the number and intensity of intercellular communications in recurrent samples relative to primary samples (Fig. 3A,B). Endothelial cells exhibited the highest number of communications, while macrophages showed the fewest (Fig. 3C). In terms of communication intensity, interactions between fibroblasts and NK cells were the strongest (Fig. 3C). Comparative analysis of communication-related genes between primary and recurrent samples identified 126 differentially expressed genes (Fig. 3D, Table A1), most of which were enriched in pathways such as cell adhesion molecules, cytokine-cytokine receptor interactions, and the PI3K-Akt pathway (Fig. 3E). Pathway enrichment analysis of the top 10 differentially expressed genes within each cell cluster revealed significant enrichment in cytokine-cytokine receptor interactions, natural killer cell-mediated cytotoxicity, and chemokine signaling pathways (Fig. 3F). Notably, chemoattractant cytokine ligand (CCL) signaling exhibited the most substantial changes in recurrent samples, particularly in endothelial cells compared to primary samples (Fig. A2).

### 3.3 Risk Score Construction Based on Relapse-Specific Genes Using the LASSO-Cox Algorithm

**Figure 4:**
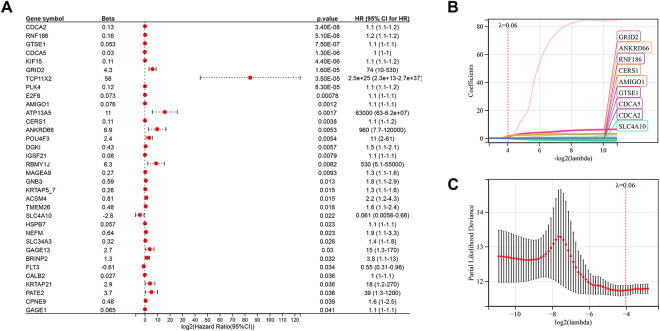

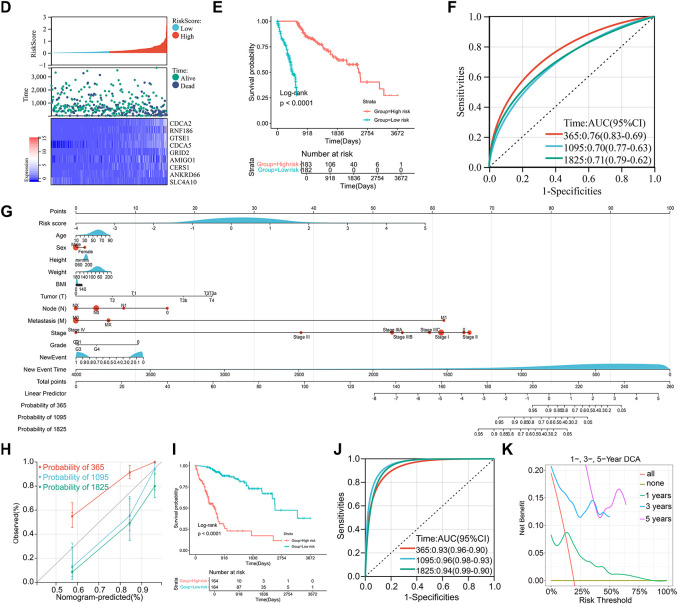
Development of a risk score model using the Least absolute shrinkage and selection operator (LASSO) algorithm and integration with clinical parameters. (**A**) Univariate Cox analysis identifying 34 prognostic genes. (**B**,**C**) Gene selection using the LASSO-Cox algorithm for constructing the optimal model. (**D**) Expression patterns and survival time relationships of 9 risk score genes. (**E**) Kaplan-Meier analysis demonstrating poorer prognosis in the high-risk group compared to the low-risk group. (**F**) Receiver operating characteristic curve (ROC) curve analysis showing Area Under Curve (AUC) values for the risk score model at 1, 3, and 5 years. (**G**) Nomogram integrating risk scores and clinical factors to predict 1-, 3-, and 5-year survival rates. (**H**) Calibration curve demonstrating the model’s accuracy in predicting patient survival rates. (**I**) Kaplan-Meier analysis indicating worse prognosis for patients with higher nomogram scores. (**J**) ROC curve showing AUC values for the nomogram scores at 1, 3, and 5 years. (**K**) Decision curve analysis to verify model effectiveness.

To identify relapse-specific genes associated with prognosis, we conducted univariate survival analysis on 180 differentially expressed genes, identifying 34 genes with significant associations (*p* < 0.05) (Fig. 4A). LASSO regression was used to select 9 key genes—CDCA2, RNF186, GTSE1, CDCA5, GRID2, AMIGO1, CERS1, ANKRD66, and SLC4A10—to construct a prognostic risk score model (Fig. 4B,C).

RiskScore = 0.0644CDCA2 + 0.0625RNF186 + 0.0035GTSE1 + 0.0001CDCA5 + 1.0301GRID2 + 0.0093AMIGO1 + 0.0148CERS1 + 1.5702ANKRD66 − 0.1633SLC4A10

Heatmap analysis indicated consistent expression patterns across most selected genes, except for CDCA5 (Fig. 4D). Kaplan-Meier survival analysis showed that the high-risk group had a significantly lower mortality rate than the low-risk group (Fig. 4E). The prognostic accuracy of the model was assessed using ROC curves, yielding AUC values of 0.76, 0.70, and 0.71 for 1-year, 3-year, and 5-year survival, respectively (Fig. 4F). A nomogram combining clinical parameters and the risk score was developed to predict 1-year, 3-year, and 5-year survival outcomes (Fig. 4G). Calibration curves confirmed the robustness of the model’s predictive capabilities (Fig. 4H). Kaplan-Meier analysis demonstrated that patients with higher nomogram scores had worse prognoses (Fig. 4I), and further ROC analysis yielded AUC values of 0.93, 0.96, and 0.94 for 1-year, 3-year, and 5-year survival, respectively (Fig. 4J). The DCA decision curve showed that the prediction of 1-year survival was good, but the performance was distorted for 3- and 5-year survival, which may be due to the low proportion of patients with survival of more than 3 and 5 years in the entire cohort (Fig. 4K).

### 3.4 Immune Landscape, Tumor Stemness, and Genomic Heterogeneity of Risk Scores

**Figure 5:**
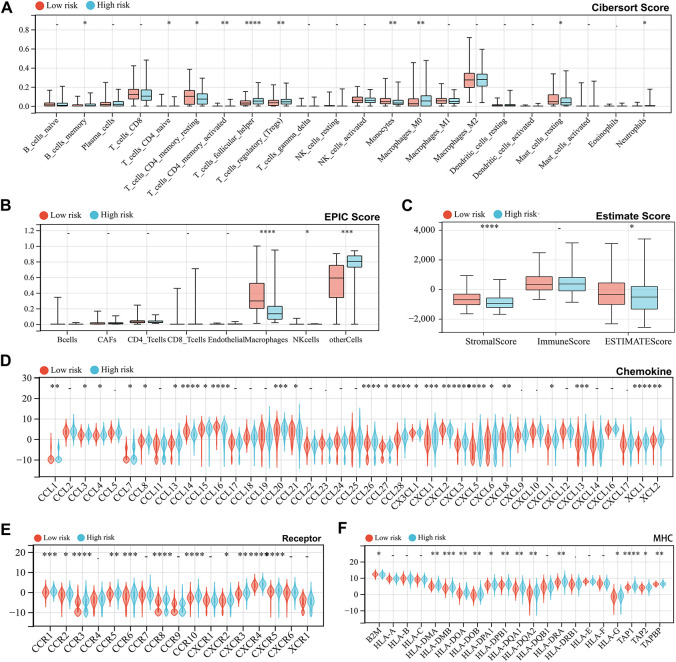

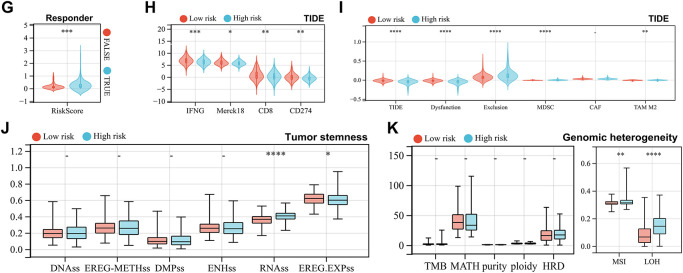
Analysis of immune status, tumor stemness, and genomic heterogeneity under risk score grouping. (**A**–**C**) Cibersort, Estimation of Proportions of Immune and Cancer cells (EPIC), and Estimate algorithms respectively calculated 22 immune cell infiltration scores (**A**), 8 immune cell infiltration scores (**B**), and overall immune scores (**C**) of high-risk and low-risk groups in the TCGA cohort samples and performed differential tests. (**D**–**F**) Differential expression of genes in three immune pathways between high-risk and low-risk groups, including chemokine (**D**), receptor (**E**), and major histocompatibility complex (MHC) (**F**). (**G**) Tumor Immune Dysfunction and Exclusion (TIDE) algorithm evaluates the difference in risk scores between the expected immunotherapy response group and the non-response group. (**H**) TIDE algorithm evaluates Interferon-γ (IFNG), Merck 18, CD8 Subunit Alpha (CD8), and CD274 scores in high-risk and low-risk groups. (**I**) TIDE algorithm evaluates TIDE, Dysfunction, Exclusion, Myeloid-derived suppressor cells (MDSC), cancer associated fibroblasts (CAF), and Tumor-Associated Macrophages (TAM) M2 scores in high-risk and low-risk groups. (**J**) Six scoring methods evaluate tumor stemness in high-risk and low-risk groups. (**K**) Seven scoring methods evaluate tumor genomic heterogeneity in high-risk and low-risk groups. *indicates *p* < 0.05, **indicates *p* < 0.01, ***indicates *p* < 0.001 and ****indicates *p* < 0.0001, -indicates no significance.

To assess the association between the risk score and immune status, we divided the TCGA-LIHC cohort into high-risk and low-risk groups. The CIBERSORT algorithm, analyzing 22 immune cell populations, revealed differences in immune cell infiltration between the groups, particularly in T cells, macrophages, and monocytes (Fig. 5A). Similarly, the EPIC algorithm showed higher macrophage infiltration in the low-risk group (Fig. 5B), while the Estimate algorithm also indicated increased immune infiltration in low-risk patients (Fig. 5C). Additionally, we extracted three categories of immune pathway genes (chemokines, receptors, and MHC) from the TCGA-LIHC cohort for comparison based on gene communication and signaling alterations. Most immune genes exhibited differential expression between the two groups. Specifically, chemokine-related genes were predominantly upregulated in the high-risk group, with a few exceptions showing downregulation (Fig. 5D), while receptor and MHC-related genes were consistently upregulated (Fig. 5E,F). Furthermore, the TIDE algorithm predicted higher risk scores in patients expected to respond to immunotherapy (Fig. 5G). High-risk patients exhibited lower scores for IFNG, Merck18, CD8, CD274, TIDE, Dysfunction, and TAM M2, but higher Exclusion and MDSC scores (Fig. 5H,I). Regarding tumor stemness, two algorithms produced conflicting results for risk groups, while other algorithms showed no significant differences (Fig. 5J). Genomic heterogeneity analysis demonstrated higher MSI and LOH scores in the high-risk group, suggesting a poor prognosis (Fig. 5K).

### 3.5 Expression and Prognostic Analysis of Risk Score Genes

**Figure 6:**
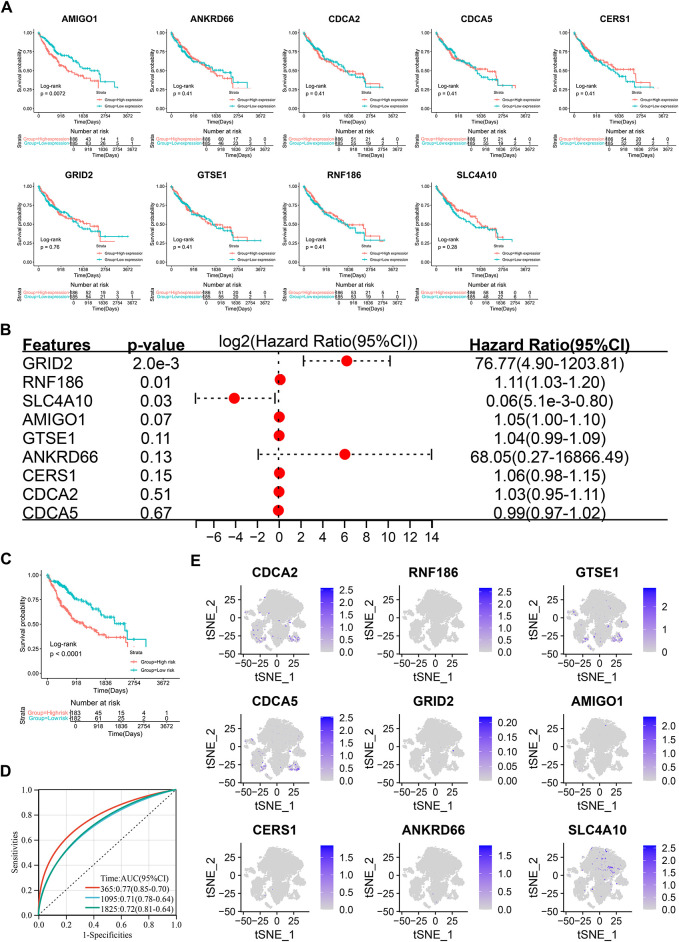
Prognostic significance and single-cell analysis of the 9 risk score genes. (**A**) Kaplan-Meier survival analysis of the 9 risk score genes. (**B**) Multivariate Cox regression analysis of the 9 risk score genes. (**C**) Kaplan-Meier survival analysis of the multivariate Cox regression risk model. (**D**) ROC curve of the multivariate Cox regression risk model. (**E**) Expression distribution of the 9 risk score genes in the single-cell t-SNE plot.

**Figure A3:**
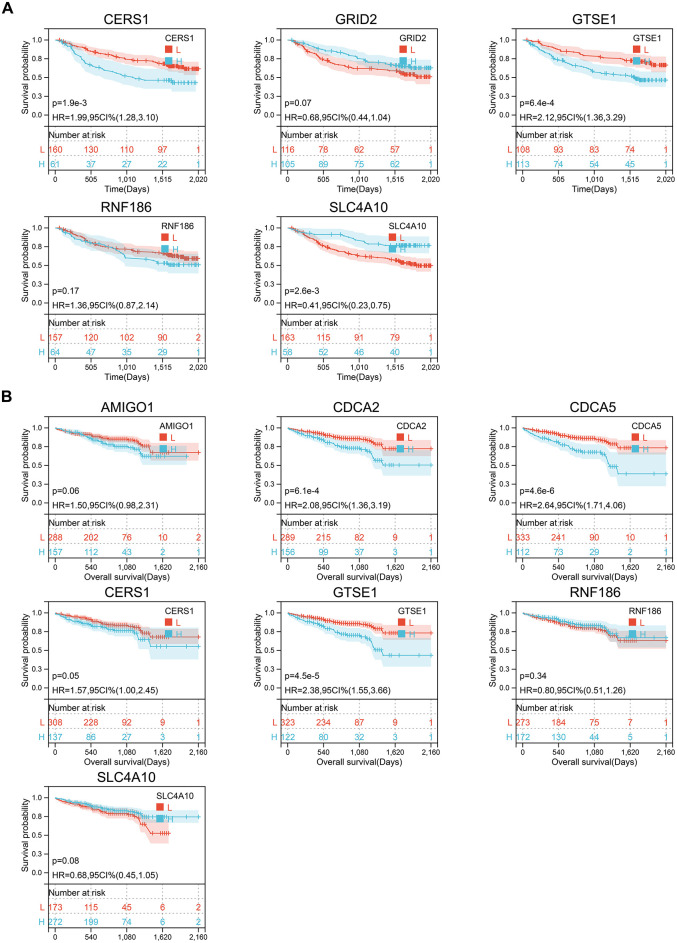
The prognostic efficacy of the risk score genes was validated in an external cohort. (**A**) Kaplan-Meier survival analysis of risk score genes in the external cohort GSE14520. (**B**) Kaplan-Meier survival analysis of risk score genes in the external cohort ICGC-LIRI-JP.

To elucidate the role of the 9 risk score genes in hepatocellular carcinoma (HCC), we performed Kaplan-Meier (KM) survival analysis to evaluate their association with patient outcomes. In the TCGA cohort, the results showed that most genes alone did not predict prognosis, with the exception of AMIGO1 (Fig. 6A). In GSE14520, the transcript levels of CERS1, GTSE1, and SLC4A10 could distinguish between different lineages, while GRID2 and RNF186 did not (Fig. A3A). In the ICGC-LIRI-JP cohort, the transcript levels of CDCA2, CDCA5, and GTSE1 were closely associated with patient prognosis, while AMIGO1, CERS1, RNF186, and SLC4A10 were not significant (Fig. A3B). Multivariate COX regression can be used to evaluate the simultaneous contribution of all included variables, while controlling for covariates was evaluated to more fully assess the prognostic role of each gene in the risk score model. Inclusion of the nine genes in multivariate COX regression analysis identified GRID2, RNF186, and SLC4A10 as independent prognostic factors (Fig. 6B). Their inclusion in a prognostic model effectively distinguished different prognostic subgroups (Fig. 6C), with ROC curve areas under the curve (AUC) for 1-, 3-, and 5-year survival predictions at 0.77, 0.71, and 0.72, respectively (Fig. 6D). A single-cell t-SNE expression heatmap revealed distinct expression patterns of the 9 genes, with SLC4A10 showing higher expression levels, while CDCA2 and CDCA5 exhibited similar expression patterns (Fig. 6E).

### 3.6 Inhibition of RNF186, but Not GRID2 or SLC4A10, Suppresses Huh7 Cell Proliferation

**Figure 7:**
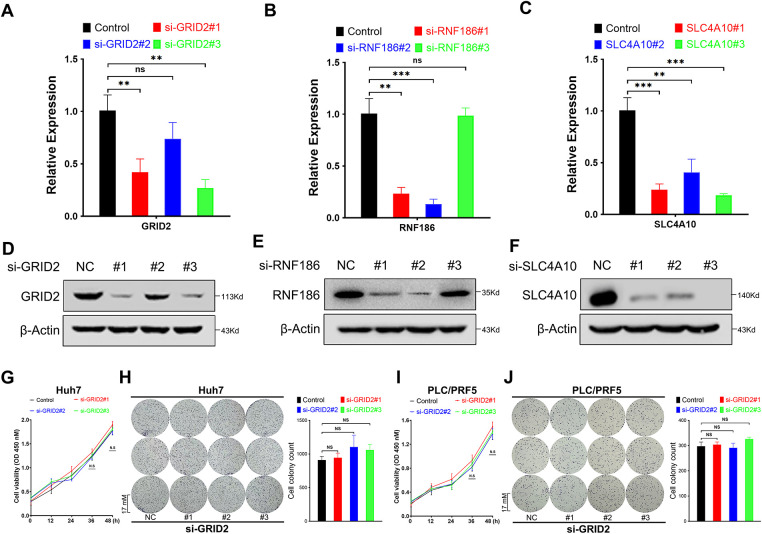

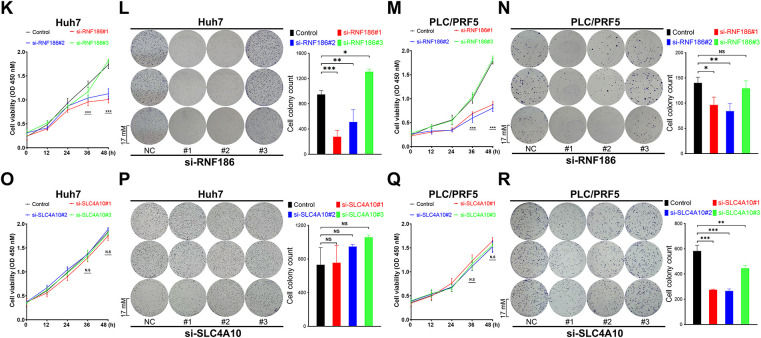
Functions of glutamate ionotropic receptor delta type subunit 2 (GRID2), ring finger protein 186 (RNF186), and solute carrier family 4 member 10 (SLC4A10) in hepatocellular carcinoma (HCC). (**A**–**C**) Real-time fluorescence quantitative polymerase chain reaction (RT-qPCR) detects the efficiency of si-RNA, including GRID2 (**A**), RNF186 (**B**), and SLC4A10 (**C**). (**D**–**F**) Western immunoblotting was used to detect the protein knockdown efficiency of si-RNA, including GRID2 (**D**), RNF186 (**E**), and SLC4A10 (**F**). (**G**,**H**) Cell counting kit-8 (CCK-8) (**G**) and colony formation assay (**H**) to detect the effect of si-GRID2 on the proliferation activity of Huh7 cells. (**I**,**J**) CCK-8 (**I**) and colony formation assay (**J**) to detect the effect of si-GRID2 on the proliferation activity of PLC/PRF/5 cells. (**K**,**L**) CCK-8 (**K**) and colony formation assay (**L**) to detect the effect of si-RNF186 on the proliferation activity of Huh7 cells. (**M**,**N**) CCK-8 (**M**) and colony formation assay (**N**) to detect the effect of si-RNF186 on the proliferation activity of PLC/PRF/5 cells. (**O**,**P**) CCK-8 (**O**) and colony formation assay (**P**) to detect the effect of si-SLC4A10 on the proliferation activity of Huh7 cells. (**Q**,**R**) CCK-8 (**Q**) and colony formation assay (**R**) to detect the effect of si-SLC4A10 on the proliferation activity of PLC/PRF/5 cells. *ns* indicates no significance, *indicates *p* < 0.05, **indicates *p* < 0.01, ***indicates *p* < 0.001.

**Figure A4:**
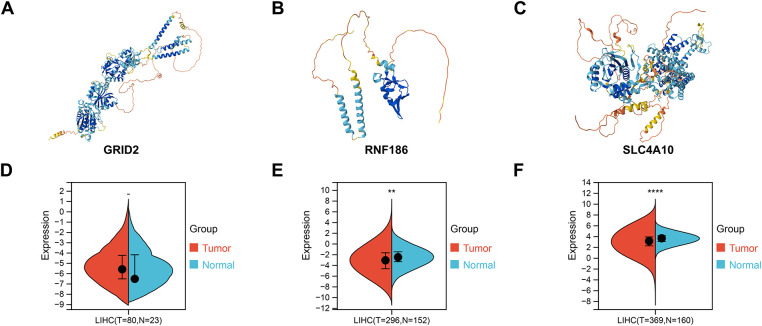
Functions of GRID2, RNF186, and SLC4A10 in hepatocellular carcinoma (HCC). (**A**) GRID2 protein structure predicted by alphafold. (**B**) RNF186 protein structure predicted by alphafold. (**C**) SLC4A10 protein structure predicted by alphafold. (**D**) Difference in GRID2 transcription levels between normal and tumor groups in the TCGA-LIHC cohort. (**E**) Difference in RNF186 transcription levels between normal and tumor groups in the TCGA-LIHC cohort. (**F**) Difference in SLC4A10 transcription levels between normal and tumor groups in the TCGA-LIHC cohort. **p < 0.01, ****p < 0.0001, -no significance.

Using Alphafold AI, we predicted the spatial conformations of GRID2, RNF186, and SLC4A10, which corresponded to proteins with molecular weights of 113 kDa, 24 kDa, and 125 kDa, respectively (Fig. A4A–C). In the TCGA cohort, RNF186 and SLC2A10 were lower expressed in tumor tissues, while GRID2 was nonsense (Fig. A4D–F). We used siRNA to suppress the expression of GRID2, RNF186, and SLC4A10, which were confirmed by real-time quantitative PCR and immunoblotting (Fig. 7A–F). Subsequent assays evaluated the proliferative capacity of Huh7 and PLC/PRF/5 hepatocellular carcinoma cells. CCK-8 and colony formation assays showed that GRID2 inhibition did not affect Huh7 and PLC/PRF/5 proliferation (Fig. 7G–J), while RNF186 inhibition significantly reduced cell proliferation (Fig. 7K–N). The results of the colony formation assay showed that si-SLC4A10#1 reduced the viability of PLC/PRF/5 cells, although this finding was not supported by Huh7 cells (Fig. 7O–R).

### 3.7 RNF186 Promotes Huh7 Cell Proliferation via SESN2

**Figure 8:**
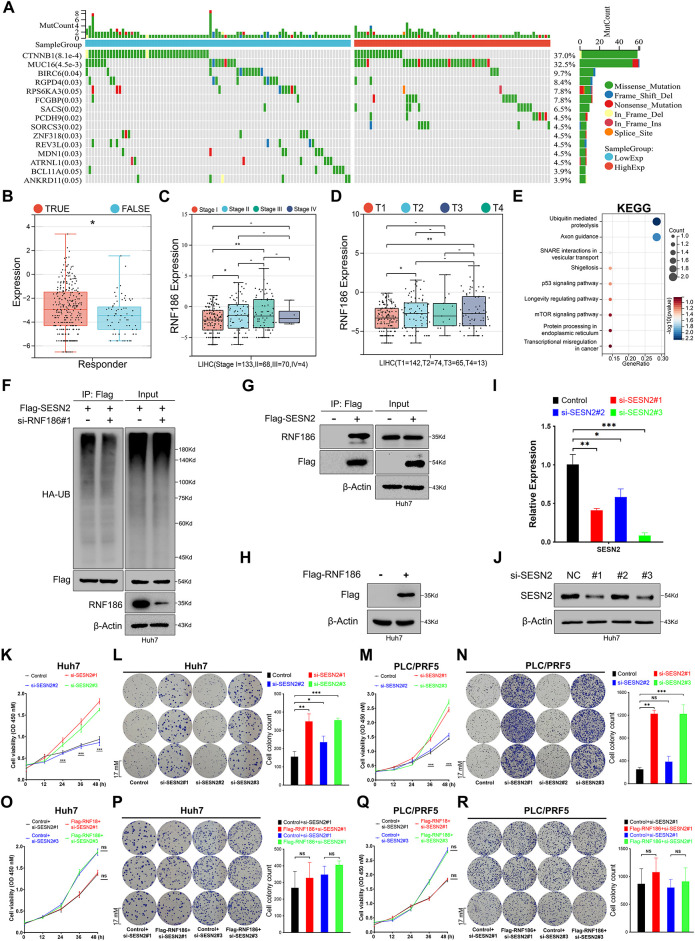
RNF186 promotes Huh7 cell proliferation in a manner dependent on SESN2. (**A**) Mutation analysis of the TCGA-LIHC cohort grouped by RNF186 transcript levels. (**B**) Evaluation of the relationship between RNF186 expression and immune response predicted by the TIDE algorithm. (**C**) Evaluation of the relationship between RNF186 expression and HCC clinical stage. (**D**) Evaluation of the relationship between RNF186 expression and whether HCC is primary or not. (**E**) KEGG enrichment analysis of the interacting protein profile of RNF186. (**F**) Huh7 cells were transfected with plasmids as indicated in the figure, lysed using the modified lysis buffer (Guan-HCl), and incubated with anti-Flag magnetic beads for 4–8 h at 4°C. Western blotting was performed using the antibodies indicated in the figure. (**G**) Huh7 cells were transfected with plasmids as indicated in the figure, lysed using the non-modified lysis buffer (RIPA), and incubated with anti-Flag magnetic beads for 4–8 h at 4°C. Western blotting was performed using the antibodies indicated in the figure. (**H**) Western blot analysis of Huh7 cells with or without overexpression of RNF186 using Flag or β-Actin antibodies. (**I**) Real-time fluorescence quantitative PCR to detect the inhibitory efficiency of si-SESN2. (**J**) Western immunoblotting detects the protein knockdown efficiency of si-SESN2. (**K**,**L**) CCK-8 (**H**) and colony formation assay (**I**) to detect the effect of si-SESN2 on the proliferation activity of Huh7 cells. (**M**,**N**) CCK-8 (**J**) and colony formation assay (**K**) to detect the effect of si-SESN2 on the proliferation activity of PLC/PRF/5 cells. (**O**,**P**) CCK-8 (**L**) and colony formation assay (**M**) were used to detect the effects of si-SESN2 transfection on the proliferation activity of Huh7 cells with or without overexpression of RNF186. (**Q**,**R**) CCK-8 (**N**) and colony formation assay (**O**) were used to detect the effects of si-SESN2 transfection on the proliferation activity of PLC/PRF/5 cells with or without overexpression of RNF186. *ns* and -indicate no significance, *indicates *p* < 0.05, **indicates *p* < 0.01, ***indicates *p* < 0.001.

**Figure A5:**
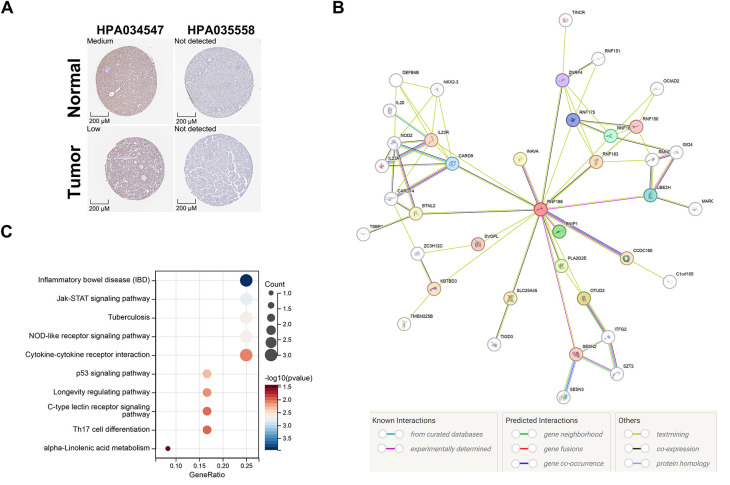

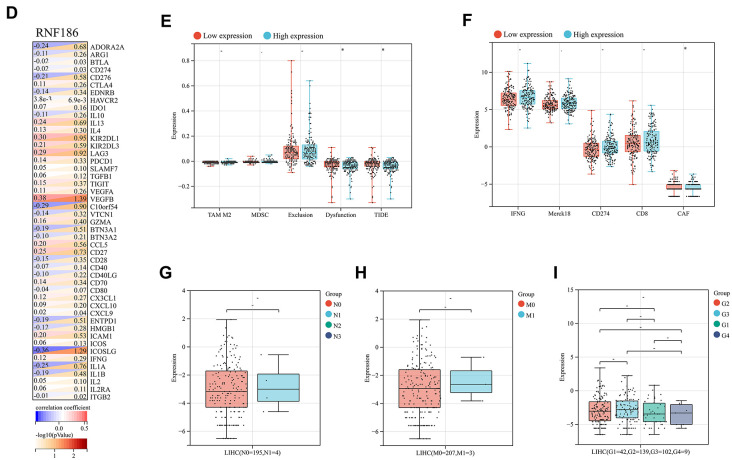
RNF186 promotes Huh7 cell proliferation in a manner dependent on SESN2. (**A**) Immunohistochemical detection of RNF186 expression in normal and HCC tissues in the HPA database. (**B**) Protein interaction network of RNF186 in the STRING database. (**C**) KEGG analysis of 40 RNF186 interacting proteins. (**D**) Correlation analysis between RNF186 expression and immune checkpoint genes. (**E**) Evaluation of RNF186 expression and tumor grade. (**F**,**G**) Differential analysis of immunotherapy scores calculated by the “TIDE” algorithm grouped by RNF186 expression. (**H**) Evaluation of the relationship between RNF186 expression and tumor lymph node metastasis. (**I**) Evaluation of the relationship between RNF186 expression and distant tumor metastasis. *p < 0.05, -no significance.

Immunohistochemistry data from the HPA database showed that there was no significant difference in RNF186 in tumor tissues compared with normal liver tissues (Fig. A5A). STRING analysis identified interactions between RNF186 and the JAK family, inflammatory bowel disease-related proteins, NOD-like receptor signaling proteins, and components of cytokine-cytokine receptor interactions (Fig. A5B,C). Mutation profiling showed that low expression of RNF186 was associated with higher mutation frequencies of genes like CTNNB1, BIRC6, and RGPD4, while high expression correlated with increased mutation frequencies of MUC16, PCDH9, and SORCS3 (Fig. 8A). Furthermore, correlation analysis suggested that RNF186 was not correlated with various immune checkpoint genes at the transcriptional level (Fig. A5D), but the transcriptional level of RNF186 was higher in the immunotherapy response group evaluated by TIDE (Fig. 8B). In addition, in the sample immunotherapy scores evaluated by the “TIDE” algorithm, Dysfunction, CAF, and TIDE scores were closely related to the expression of RNF186 (Fig. A5E,F). Not only that, but RNF186 expression was also closely related to the stage of HCC and whether it was primary (Fig. 8C,D), although it was not related to tumor lymph node metastasis, whether it was distant metastasis, and tumor grade (Fig. A5G–I).

To investigate potential downstream targets of RNF186 in cell proliferation regulation, we used BioGrid to identify interacting proteins. KEGG pathway enrichment analysis of these interacting proteins revealed significant enrichment in proteasomal degradation and ubiquitination pathways. Notably, among the enriched pathways, the crossover protein between the p53 and mTOR signaling pathways is SESN2 (Fig. 8E), suggesting that RNF186 may regulate cell proliferation in an SESN2-dependent manner [25,26]. To test this hypothesis, ubiquitination experiments in Huh7 cells showed that inhibition of RNF186 reduced the ubiquitination modification of SESN2 (Fig. 8F). Furthermore, exogenous SESN2 protein successfully co-immunoprecipitated endogenous RNF186 from Huh7 cells (Fig. 8G). We modulated SESN2 and RNF186 expression using siRNA and Flag-RNF186 plasmids, respectively, with RT-qPCR and immunoblotting confirming the efficacy of these interventions (Fig. 8H–J). Cell proliferation was assessed in Huh7 and PCL/PRF/5 cells using CCK-8 and colony formation assays. SESN2 knockdown significantly enhanced the proliferation of both cell lines (Fig. 8K–N). Furthermore, under SESN2 knockdown conditions, Flag-RNF186 overexpression failed to promote cell proliferation in either Huh7 or PCL/PRF/5 cells (Fig. 8O–R), indicating that SESN2 suppression effectively abrogates the proliferative effects of RNF186 in Huh7 cells.

## 4 Discussion

In this study, we conducted an in-depth analysis of single-cell sequencing data from liver cancer samples, focusing on the identification and functional characterization of genes associated with recurrence. We selected high-quality cells, clustered them into 18 distinct populations, and annotated seven major cell types. Notably, endothelial cells and macrophages emerged as key cell clusters linked to recurrence. We further developed a prognostic model based on macrophage-specific genes using the LASSO-Cox algorithm, demonstrating significant correlations between risk scores, immune landscapes, and patient outcomes. Multivariate Cox regression and functional assays identified GRID2, RNF186, and SLC4A10 as pivotal genes, with RNF186 shown to promote Huh7 cell proliferation in an SESN2-dependent manner.

While previous studies have primarily focused on various immune subsets, our work expands this knowledge by identifying specific cell clusters and prognostic genes associated with recurrence in liver cancer [11,27,28]. Macrophages and endothelial cells were identified as hallmark clusters in recurrent patients. Indeed, macrophages play a crucial role in liver cancer progression and recurrence [29–31]. Specifically, a reduction in macrophage populations in recurrent samples could reflect a diminished immune surveillance capacity, favoring tumor immune evasion [32,33]. It is particularly important to note that the subtype composition of macrophages may also shift. M1-like macrophages, typically associated with anti-tumor activity, may decrease, while M2-like macrophages, which promote tumor progression through immunosuppressive cytokine secretion and tissue remodeling, may be enriched [34].

Endothelial cell dysfunction, on the other hand, is a key driver of tumor angiogenesis and vascular remodeling, which are critical processes for sustaining tumor growth and metastasis [11,35]. An increase in endothelial cells in recurrent samples may reflect enhanced angiogenic activity, contributing to a pro-recurrence microenvironment [30]. Moreover, endothelial cells also influence the tumor immune microenvironment by modulating immune cell trafficking and response to immunotherapy [36]. Their enrichment in recurrent samples underscores their role in shaping tumor progression and resistance to therapeutic interventions [30,37]. Additionally, chemokine-mediated cell communication was notably altered in these clusters in recurrent samples, suggesting immune microenvironment changes in recurrent tumors. Although chemokines are implicated in tumor progression and immune evasion [37,38], their role in HCC recurrence remains poorly understood [39].

Multiple immune deconvolution algorithms were used to ensure the robustness of immune infiltration assessment. Although CIBERSORT, EPIC, and ESTIMATE rely on distinct reference matrices and computational principles, the major immune infiltration patterns identified by these methods were largely consistent. Minor discrepancies likely reflect methodological differences rather than biological contradictions. Considering that each algorithm captures unique aspects of the tumor microenvironment, we adopted a complementary strategy to interpret convergent trends rather than combine absolute values. The findings reveal a complex immune and genomic landscape in high-risk patients. Despite lower scores for key immunotherapy-related markers such as IFNG, CD8, CD274, and TIDE, these patients demonstrate a paradoxical susceptibility to immunotherapy. This discrepancy may be attributed to the interplay between tumor microenvironment features and genomic heterogeneity [40,41]. High-risk patients showed elevated Exclusion and MDSC scores, indicating an immune-suppressive microenvironment, which could be offset by other factors such as increased tumor mutational burden (TMB), microsatellite instability (MSI), or loss of heterozygosity (LOH) [42,43]. These genomic alterations may enhance the presentation of neoantigens, thereby improving immune recognition and response to immune checkpoint blockade, despite low baseline expression of classical markers like CD274 [44,45]. Enhanced tumor stemness can lead to enhanced tumor immune evasion [46,47]. In our analysis, however, different tumor stemness algorithms produced inconsistent results, with four out of six algorithms showing no significant differences. These discrepancies may arise from the distinct methodological principles of each algorithm. For instance, the RNAss and EREG.EXPss scores are calculated based on different molecular profiles, including RNA expression and methylation signatures, which may capture different aspects of tumor biology. The conflicting results from tumor stemness algorithms also highlight the complexity of the tumor’s biological behavior and its interaction with the immune system. Further exploration of these mechanisms, particularly the relationship between genomic instability and immunotherapy response, is warranted to clarify these findings and optimize therapeutic strategies for high-risk patients.

RNF186, an E3 ubiquitin ligase, is implicated in various diseases, such as ulcerative colitis and bladder cancer, by modulating substrate ubiquitination [48,49]. This study identified RNF186 as a signature gene for HCC recurrence and confirmed its pro-proliferative effect through cell function experiments, which is dependent on the ubiquitination regulation of SESN2. Regarding HCC recurrence, we tend to support the view that RNF186 maintains the proliferative capacity of HCC cells, which supports the recurrence of HCC, because in other diseases, genomic mutations in RNF186 itself often lead to the onset of the disease [48,50]. Conversely, there is also evidence that RNF186 is regulated by other molecules to promote cancer progression [49]. Human macrophages with the RNF186-A64T variant exhibit impaired NOD2 induction, reducing innate immune responses [51], suggesting that RNF186 dysregulation may affect macrophage function and contribute to HCC recurrence. Low RNF186 expression correlates with higher mutation frequencies in CTNNB1, BIRC6, and RGPD4, suggesting its potential role in suppressing Wnt/β-catenin signaling, maintaining apoptosis regulation, and preserving genomic stability [52,53]. Conversely, high RNF186 expression associates with increased mutations in MUC16, PCDH9, and SORCS3, which may indicate distinct tumor subtypes with unique molecular dependencies, such as immune evasion, disrupted tumor suppressor functions, and altered neurotrophin signaling [54,55]. These findings highlight the dual role of RNF186 in influencing HCC recurrence and immunotherapy relevance, underscoring its potential as a biomarker or therapeutic target for HCC.

Despite these significant findings, several limitations must be acknowledged. For example, although 18 samples (12 primary samples and 6 recurrent samples) were included in this study, the sample number was still small for hepatocellular carcinoma, which may lead to abnormal results that are caused by the heterogeneity of hepatocellular carcinoma. The recurrence prediction model in this study is based on scRNA-seq data, but the high cost and complex analysis limit its clinical application. To improve feasibility, key genes can be validated by more economical detection methods (such as qPCR or immunohistochemistry) in the future, and the stability of the model can be further verified in independent cohorts. In addition, combining other clinical parameters to optimize the model will help to achieve its wide application in personalized treatment, and its clinical utility requires evaluation through prospective studies. Moreover, additional *in vivo* and *in vitro* mechanistic studies are necessary to elucidate the precise roles of GRID2, RNF186, and SLC4A10 in liver cancer.

## 5 Conclusion

This study provides a comprehensive analysis of recurrence-related cell clusters and genes in liver cancer, revealing their prognostic significance and therapeutic potential. Functional experiments demonstrate that RNF186 promotes cell proliferation in an SESN2-dependent manner, laying a foundation for future research into the tumor microenvironment and the discovery of novel therapeutic targets for liver cancer.

## Data Availability

The datasets presented in this study can be found in online repositories, which deposited into the CNGB Sequence Archive (CNSA) of the China National GeneBank DataBase (CNGBdb, https://db.cngb.org/cnsa/) with accession number CNP0000650. Bulk RNA data comes from the TCGA project. All public data are fully described in the methods section.

## Appendix A

**Table A1:** Differential gene analysis.

Gene	*P val*	Avg_log2FC	Pct.1	Pct.2	*P*_val_adj	Cluster
SLC4A10	6.26 × 10^−100^	2.27488	0.396	0.223	1.23 × 10^−95^	0
LTK	1.57 × 10^−29^	2.18877	0.096	0.044	3.09 × 10^−25^	0
IL26	1.79 × 10^−12^	2.51119	0.033	0.014	3.53 × 10^−8^	0
WNT7A	1.35 × 10^−11^	2.6382	0.027	0.011	2.65 × 10^−7^	0
IL22	2.01 × 10^−7^	2.30399	0.026	0.013	3.95 × 10^−3^	0
C12orf40	1.96 × 10^−6^	2.40221	0.025	0.013	3.86 × 10^−2^	0
AMIGO1	2.48 × 10^−6^	2.10931	0.032	0.018	4.89 × 10^−2^	0
UNC45B	2.60 × 10^−6^	2.0701	0.034	0.019	5.13 × 10^−2^	0
KRTAP5-7	8.86 × 10^−5^	2.39987	0.011	0.005	1	0
IL17F	0.001331	2.89805	0.019	0.011	1	0
RP11-277P12.6	0	4.1608	0.726	0.252	0	1
KIR2DL3	0	4.30391	0.615	0.189	0	1
KIR3DL1	0	4.44638	0.564	0.151	0	1
KIR2DL1	0	4.62762	0.516	0.127	0	1
KIR3DL2	0	4.4834	0.518	0.145	0	1
KIR3DL3	0	4.85949	0.355	0.045	0	1
NLRP7	8.45 × 10^−19^	4.04772	0.035	0.011	1.66 × 10^−14^	1
PRSS57	1.35 × 10^−9^	4.35164	0.016	0.005	2.66 × 10^−5^	1
EPYC	2.48 × 10^−8^	4.293	0.018	0.006	4.88 × 10^−4^	1
ZMAT4	0.0027221	4.13486	0.014	0.007	1	1
TRAT1	4.09 × 10^−135^	1.27612	0.949	0.779	8.05 × 10^−131^	2
GFPT2	1.52 × 10^−7^	1.27978	0.17	0.128	3.00 × 10^−3^	2
ST6GALNAC1	4.28 × 10^−6^	2.14406	0.016	0.006	8.42 × 10^−2^	2
CCL1	7.06 × 10^−6^	2.20308	0.01	0.003	1.39 × 10^−^1	2
MBOAT4	7.95 × 10^−5^	1.83741	0.057	0.038	1	2
KRTAP5-7	7.99 × 10^−5^	1.61294	0.013	0.005	1	2
KRT2	0.0003679	1.9076	0.015	0.007	1	2
NEFM	0.001337	1.2809	0.045	0.031	1	2
WNT7A	0.0030211	1.83001	0.021	0.013	1	2
COL10A1	0.0040608	1.45662	0.014	0.007	1	2
VCY1B	1.33 × 10^−213^	5.59854	0.103	0.005	2.61 × 10^−209^	3
CBLN4	4.97 × 10^−205^	5.63295	0.087	0.003	9.79 × 10^−201^	3
OCA2	1.83 × 10^−177^	5.86642	0.111	0.009	3.61 × 10^−173^	3
OR10J5	4.42 × 10^−35^	6.23819	0.014	0	8.69 × 10^−31^	3
RNF186	9.51 × 10^−27^	6.02052	0.014	0.001	1.87 × 10^−22^	3
GDPD4	2.67 × 10^−26^	5.84972	0.016	0.001	5.25 × 10^−22^	3
CLDN9	2.62 × 10^−23^	5.91591	0.014	0.001	5.16 × 10^−19^	3
RBMY1B	1.90 × 10^−22^	5.86373	0.011	0.001	3.75 × 10^−18^	3
RBMY1J	2.07 × 10^−22^	5.67885	0.01	0	4.08 × 10^−18^	3
UBE2U	8.27 × 10^−19^	5.65243	0.011	0.001	1.63 × 10^−14^	3
CD1E	2.74 × 10^−233^	4.20601	0.662	0.312	1.23 × 10^−95^	4
CD1B	2.37 × 10^−232^	4.26644	0.481	0.151	3.09 × 10^−25^	4
FLT3	1.54 × 10^−144^	5.1036	0.277	0.076	3.53 × 10^−8^	4
CACNA2D3	1.50 × 10^−62^	4.24492	0.167	0.054	2.65 × 10^−7^	4
CLEC4F	1.57 × 10^−41^	4.95046	0.104	0.032	3.95 × 10^−3^	4
XCR1	3.12 × 10^−28^	4.68302	0.071	0.022	3.86 × 10^−2^	4
LIPN	2.44 × 10^−22^	4.44353	0.036	0.008	4.89 × 10^−2^	4
CD207	8.58 × 10^−22^	5.31131	0.085	0.033	5.13 × 10^−2^	4
PPM1J	5.29 × 10^−15^	4.74916	0.036	0.011	1	4
CCNA1	3.11 × 10^−6^	4.17339	0.013	0.004	1	4
FOXP3	3.16 × 10^−287^	4.33154	0.533	0.159	0	5
CCR8	1.57 × 10^−244^	4.48931	0.537	0.185	0	5
GNG8	2.35 × 10^−92^	4.06267	0.155	0.034	0	5
CD177	2.81 × 10^−37^	4.40031	0.157	0.064	0	5
PVALB	8.20 × 10^−29^	3.94647	0.074	0.022	0	5
AHSP	1.74 × 10^−22^	4.15447	0.077	0.028	0	5
CD300LD	9.87 × 10^−14^	4.69525	0.039	0.013	1.66 × 10^−14^	5
ARX	2.27 × 10^−11^	5.21748	0.014	0.002	2.66 × 10^−5^	5
DGKI	1.01 × 10^−8^	3.86791	0.037	0.015	4.88 × 10^−4^	5
MYBPC2	2.64 × 10^−6^	3.93218	0.011	0.003	1	5
IGSF21	2.03 × 10^−158^	3.92451	0.268	0.056	8.05 × 10^−131^	6
TMEM26	2.74 × 10^−127^	3.82477	0.195	0.036	3.00 × 10^−3^	6
HS3ST2	2.66 × 10^−76^	3.94625	0.13	0.026	8.42 × 10^−2^	6
CCL24	9.27 × 10^−72^	4.28147	0.091	0.014	1.39 × 10^−1^	6
PKD2L1	1.64 × 10^−61^	4.31082	0.094	0.017	1	6
TNFSF18	3.96 × 10^−13^	3.91959	0.042	0.014	1	6
SPINK6	7.46 × 10^−12^	4.94807	0.011	0.001	1	6
ANKRD66	1.35 × 10^−9^	4.82224	0.018	0.004	1	6
DUSP27	1.11 × 10^−6^	4.31534	0.01	0.002	1	6
ACSM4	7.00 × 10^−6^	3.87344	0.01	0.002	1	6
PAX5	0	6.97256	0.504	0.075	2.61 × 10^−209^	7
FCRL1	1.64 × 10^−269^	6.30058	0.502	0.119	9.79 × 10^−201^	7
CD19	1.56 × 10^−250^	6.06609	0.494	0.123	3.61 × 10^−173^	7
FCRLA	1.61 × 10^−223^	6.14524	0.539	0.168	8.69 × 10^−31^	7
KHDRBS2	3.87 × 10^−51^	5.79604	0.113	0.026	1.87 × 10^−22^	7
KLHL14	7.91 × 10^−46^	7.12408	0.091	0.019	5.25 × 10^−22^	7
KCNH8	4.06 × 10^−28^	5.7496	0.073	0.019	5.16 × 10^−19^	7
C12orf74	2.48 × 10^−23^	6.44353	0.039	0.007	3.75 × 10^−18^	7
FCRL4	9.00 × 10^−10^	6.27934	0.019	0.004	4.08 × 10^−18^	7
LCN8	4.08 × 10^−9^	5.97332	0.014	0.003	1.63 × 10^−14^	7
IL20	9.89 × 10^−10^	3.72031	0.018	0.003	1.23 × 10^−95^	8
CERS1	3.10 × 10^−8^	3.93585	0.017	0.003	3.09 × 10^−25^	8
NEUROD4	1.36 × 10^−7^	7.80849	0.012	0.002	3.53 × 10^−8^	8
CPNE9	2.02 × 10^−7^	3.48566	0.027	0.008	2.65 × 10^−7^	8
OCSTAMP	4.12 × 10^−7^	5.90075	0.014	0.003	3.95 × 10^−3^	8
PRPH	6.22 × 10^−7^	5.74714	0.012	0.002	3.86 × 10^−2^	8
SHC3	8.99 × 10^−7^	3.35554	0.026	0.008	4.89 × 10^−2^	8
RHCG	2.66 × 10^−5^	3.46443	0.026	0.009	5.13 × 10^−2^	8
ADGRB3	0.0007539	4.55255	0.011	0.003	1	8
C2orf16	0.0012103	3.84259	0.014	0.005	1	8
KIF15	1.62 × 10^−167^	4.52638	0.502	0.142	0	9
CDCA5	2.09 × 10^−156^	4.57741	0.42	0.105	0	9
GTSE1	2.02 × 10^−138^	4.39905	0.529	0.184	0	9
CDCA2	6.99 × 10^−129^	4.41556	0.377	0.096	0	9
PLK4	1.74 × 10^−124^	4.40362	0.301	0.064	0	9
HIST1H3G	3.51 × 10^−92^	5.06767	0.148	0.021	0	9
E2F8	2.60 × 10^−80^	4.59759	0.207	0.044	1.66 × 10^−14^	9
SYCE2	1.14 × 10^−42^	4.37032	0.096	0.018	2.66 × 10^−5^	9
GNB3	1.01 × 10^−11^	4.3892	0.025	0.005	4.88 × 10^−4^	9
LRIT3	5.95 × 10^−11^	5.41785	0.011	0.001	1	9
AC109829.1	4.09 × 10^−72^	6.58454	0.042	0.001	8.05 × 10^−131^	10
SPACA7	5.54 × 10^−68^	6.96228	0.034	0.001	3.00 × 10^−3^	10
C11orf97	1.65 × 10^−45^	8.79189	0.016	0	8.42 × 10^−2^	10
PRSS51	7.04 × 10^−21^	6.8627	0.018	0.001	1.39 × 10^−1^	10
CT47A10	4.95 × 10^−20^	6.18936	0.01	0	1	10
KLK4	4.99 × 10^−20^	7.58343	0.018	0.001	1	10
LRFN5	4.50 × 10^−16^	6.82028	0.022	0.002	1	10
AC131097.4	6.36 × 10^−16^	5.9679	0.014	0.001	1	10
SYT8	2.73 × 10^−9^	6.26022	0.012	0.001	1	10
HSD3B2	3.00 × 10^−8^	6.12074	0.01	0.001	1	10
POU4F3	4.47 × 10^−16^	5.03488	0.011	0	2.61 × 10^−209^	11
CALB2	2.27 × 10^−13^	4.29152	0.032	0.005	9.79 × 10^−201^	11
GATA1	3.84 × 10^−10^	4.20094	0.017	0.002	3.61 × 10^−173^	11
SERPINB3	1.13 × 10^−9^	4.48446	0.013	0.001	8.69 × 10^−31^	11
OR10H1	2.06 × 10^−9^	6.30405	0.011	0.001	1.87 × 10^−22^	11
GCM2	1.73 × 10^−8^	4.46023	0.011	0.001	5.25 × 10^−22^	11
HRASLS5	8.24 × 10^−7^	4.25864	0.011	0.001	5.16 × 10^−19^	11
MUC5AC	8.52 × 10^−7^	4.64985	0.013	0.002	3.75 × 10^−18^	11
UBQLNL	0.000152	4.31163	0.019	0.006	4.08 × 10^−18^	11
FOXL2	0.004164	5.56818	0.011	0.003	1.63 × 10^−14^	11
KRT82	1.36 × 10^−16^	3.18133	0.061	0.014	1.23 × 10^−95^	12
OR5B3	1.41 × 10^−15^	3.31509	0.035	0.005	3.09 × 10^−25^	12
PIANP	1.75 × 10^−6^	3.8333	0.015	0.003	3.53 × 10^−8^	12
CDC42BPG	1.05 × 10^−5^	3.18399	0.02	0.005	2.65 × 10^−7^	12
MAP6	0.0004949	4.32855	0.02	0.006	3.95 × 10^−3^	12
GJA3	0.0006685	3.28024	0.018	0.005	3.86 × 10^−2^	12
NCAN	0.0017926	4.20579	0.011	0.003	4.89 × 10^−2^	12
TRPM1	0.0022167	3.19966	0.063	0.037	5.13 × 10^−2^	12
FNDC11	0.0025253	4.98432	0.011	0.003	1	12
MYH15	0.0064381	4.25786	0.011	0.003	1	12
PAX4	2.93 × 10^−125^	7.83822	0.092	0.003	0	13
PDIA2	4.29 × 10^−94^	6.80038	0.051	0.001	0	13
KRTAP3-1	3.61 × 10^−40^	7.1002	0.024	0.001	0	13
DEFB103B	1.47 × 10^−27^	7.01878	0.012	0	0	13
SLC34A3	7.71 × 10^−22^	6.7498	0.017	0.001	0	13
DEFB103A	1.92 × 10^−20^	7.20853	0.01	0	0	13
OR5A1	9.30 × 10^−17^	7.49096	0.012	0	1.66 × 10^−14^	13
TKTL2	1.26 × 10^−11^	7.46493	0.017	0.002	2.66 × 10^−5^	13
TCP11X2	2.96 × 10^−11^	7.15907	0.017	0.002	4.88 × 10^−4^	13
NEUROD6	0.0001387	7.53163	0.01	0.002	1	13
SMOC2	6.36 × 10^−210^	8.24297	0.335	0.021	8.05 × 10^−131^	14
SOX17	2.02 × 10^−186^	8.58517	0.216	0.009	3.00 × 10^−3^	14
FOXF1	2.02 × 10^−161^	8.80806	0.237	0.013	8.42 × 10^−2^	14
MYOCD	2.98 × 10^−66^	8.67703	0.114	0.008	1.39 × 10^−1^	14
LRRC10B	3.77 × 10^−58^	8.54747	0.076	0.004	1	14
KRTAP21-2	7.08 × 10^−47^	8.77569	0.013	0	1	14
JPH2	1.43 × 10^−41^	8.83149	0.072	0.005	1	14
FOXF2	1.20 × 10^−21^	8.45481	0.038	0.003	1	14
HSPB7	1.79 × 10^−17^	8.30385	0.034	0.003	1	14
GABRG1	6.41 × 10^−11^	8.56627	0.021	0.002	1	14
GAGE12C	0	8.89449	0.991	0.025	2.61 × 10^−209^	15
GAGE12G	0	8.92961	0.987	0.024	9.79 × 10^−201^	15
GAGE12J	0	8.96901	0.983	0.024	3.61 × 10^−173^	15
GAGE12D	0	8.88434	0.983	0.025	8.69 × 10^−31^	15
GAGE12F	0	8.90395	0.983	0.026	1.87 × 10^−22^	15
GAGE10	0	8.78936	0.974	0.018	5.25 × 10^−22^	15
GAGE13	0	8.6895	0.983	0.028	5.16 × 10^−19^	15
GAGE1	0	8.78731	0.987	0.044	3.75 × 10^−18^	15
GAGE2E	0	9.31589	0.952	0.016	4.08 × 10^−18^	15
MAGEA9	7.93 × 10^−163^	8.61009	0.091	0.001	1.63 × 10^−14^	15
KCNQ4	2.65 × 10^−13^	6.83496	0.045	0.003	1.23 × 10^−95^	16
IFNA10	1.06 × 10^−10^	6.01285	0.018	0.001	3.09 × 10^−25^	16
C2orf57	2.37 × 10^−9^	6.27777	0.018	0.001	3.53 × 10^−8^	16
FAM43B	6.89 × 10^−8^	6.31855	0.018	0.001	2.65 × 10^−7^	16
VSTM2L	9.28 × 10^−8^	6.36233	0.027	0.002	3.95 × 10^−3^	16
CHAT	1.70 × 10^−7^	6.29001	0.018	0.001	3.86 × 10^−2^	16
SLC17A6	2.76 × 10^−6^	6.88202	0.018	0.001	4.89 × 10^−2^	16
ATP13A5	2.88 × 10^−6^	6.34684	0.018	0.001	5.13 × 10^−2^	16
PRR22	7.45 × 10^−6^	5.70779	0.027	0.003	1	16
GRID2	0.002056	5.92001	0.018	0.003	1	16
BRINP2	9.94 × 10^−58^	8.52739	0.082	0.002	0	17
GPRC6A	7.09 × 10^−35^	8.34312	0.041	0.001	0	17
ATP2B3	8.53 × 10^−15^	7.8142	0.041	0.002	0	17
HSPB3	1.58 × 10^−13^	9.00368	0.01	0	0	17
NR2E1	9.14 × 10^−11^	7.94249	0.031	0.002	0	17
ASIC5	2.10 × 10^−10^	8.33298	0.01	0	0	17
AC124312.1	2.10 × 10^−10^	7.87931	0.01	0	1.66 × 10^−14^	17
SLC18A3	3.98 × 10^−5^	8.40139	0.01	0	2.66 × 10^−5^	17
LRRC71	0.0001071	8.08189	0.01	0.001	4.88 × 10^−4^	17
PATE2	0.0008554	8.22019	0.01	0.001	1	17
